# Sugar Beet Pectin Supplementation Did Not Alter Profiles of Fecal Microbiota and Exhaled Breath in Healthy Young Adults and Healthy Elderly

**DOI:** 10.3390/nu11092193

**Published:** 2019-09-12

**Authors:** Ran An, Ellen Wilms, Agnieszka Smolinska, Gerben D.A. Hermes, Ad A.M. Masclee, Paul de Vos, Henk A. Schols, Frederik J. van Schooten, Hauke Smidt, Daisy M.A.E. Jonkers, Erwin G. Zoetendal, Freddy J. Troost

**Affiliations:** 1 Laboratory of Microbiology, Wageningen University & Research, 6708 WE Wageningen, The Netherlands; 2 Division Gastroenterology-Hepatology, Department of Internal Medicine, NUTRIM School of Nutrition and Translational Research in Metabolism, Maastricht University Medical Centre+, 6202 AZ Maastricht, The Netherlands; 3 TI Food and Nutrition, 6700 AN Wageningen, The Netherlands; 4 Department of Pharmacology and Toxicology, NUTRIM School of Nutrition and Translational Research in Metabolism, Maastricht University, 6229 ER Maastricht, The Netherlands; 5 Division of Medical Biology, Department of Pathology and Medical Biology, University of Groningen and University Medical Centre Groningen, 9713 GZ Groningen, The Netherlands; 6 Laboratory of Food Chemistry, Wageningen University & Research, 6708 WG Wageningen, The Netherlands; 7 Food Innovation and Health, Centre for Healthy Eating and Food Innovation, NUTRIM School of Nutrition and Translational Research in Metabolism, Maastricht University, 6200 MD Venlo, The Netherlands

**Keywords:** microbiota, exhaled air, dietary fiber, pectin, aging, elderly, young adults

## Abstract

Aging is accompanied with increased frailty and comorbidities, which is potentially associated with microbiome perturbations. Dietary fibers could contribute to healthy aging by beneficially impacting gut microbiota and metabolite profiles. We aimed to compare young adults with elderly and investigate the effect of pectin supplementation on fecal microbiota composition, short chain fatty acids (SCFAs), and exhaled volatile organic compounds (VOCs) while using a randomized, double-blind, placebo-controlled parallel design. Fifty-two young adults and 48 elderly consumed 15 g/day sugar beet pectin or maltodextrin for four weeks. Fecal and exhaled breath samples were collected before and after the intervention period. Fecal samples were used for microbiota profiling by 16S rRNA gene amplicon sequencing, and for analysis of SCFAs by gas chromatography (GC). Breath was used for VOC analysis by GC-tof-MS. Young adults and elderly showed similar fecal SCFA and exhaled VOC profiles. Additionally, fecal microbiota profiles were similar, with five genera significantly different in relative abundance. Pectin supplementation did not significantly alter fecal microbiota, SCFA or exhaled VOC profiles in elderly or young adults. In conclusion, aside from some minor differences in microbial composition, healthy elderly and young adults showed comparable fecal microbiota composition and activity, which were not altered by pectin supplementation.

## 1. Introduction

In line with the rising life expectancy, the aging population is increasing globally, leading to an increase in direct and indirect healthcare costs [1,2]. General health status may decline with aging and it has been associated with changes in gastrointestinal (GI) tract microbiome characteristics; e.g., changes in microbial diversity, microbiota composition, as well as microbiota function [3]. On the other hand, a substantial group of elderly is capable of maintaining the functional ability that supports wellbeing, which is defined as “healthy aging” [4]. Various studies investigated the effect of age on microbiota composition by comparing the microbiota of healthy elderly and healthy young adults. Nevertheless, the definition of “healthy” and of age cut offs used for elderly varies between studies. Mueller et al. demonstrated a lower relative abundance of *Bifidobacterium* and a higher relative abundance of enterobacteria in the elderly in four European study populations (France, Germany, Italy, and Sweden) [5]. In contrast, increased levels of *Bifidobacterium* in the microbiota of higher-aged individuals (i.e., centenarians) has also been reported, as compared to that of young adults [6]. Furthermore, the microbiota of non-institutionalized elderly had lower abundance of genes coding for carbohydrate metabolism, but increased proteolytic potential (increased abundance of genes coding for the degradation of branched-chain amino acids) when compared with that of young adults [7]. Reported alterations in microbiota composition and/or activities could in part be attributed to changes in alterations with respect to nutritional factors [8]. The intake of dietary fibers, such as the non-digestible carbohydrates fructo-oligosaccharides (FOS) [9], galacto-oligosaccharides (GOS) [10,11,12], and resistant starch, has been shown to beneficially impact intestinal microbiota composition. Supplemented non-digestible carbohydrates that reach the colon are fermented by microbes, and thereby contribute to the production of metabolites, including short chain fatty acids (SCFAs), which are known for their health promoting effects [13].

Pectin is an important member of dietary fiber that is present in many fruits, vegetables, and legumes. Pectin supplementation has been shown to affect microbiota composition both in vitro [14,15] and in vivo in rats [16,17,18], mice [19], piglets [20], but also in humans [21,22], specifically in patients with active ulcerative colitis [21] and adults with slow-transit constipation [22], although the specific effects depend on the solubility and chemical fine structure of supplemented pectin. For instance, pectin supplementation increased the relative abundance of *Bifidobacterium* when compared with controls in an in vitro study [23] as well as in adults with slow-transit constipation [22], but not in vivo in piglets [20] or rat [17], or in patients with active ulcerative colitis [21]. When considering the impact of pectin supplementation on microbial activity, most studies have focused on fecal SCFA levels, although it should be noted that the majority of the metabolites are absorbed in the intestine. Currently published studies have been reported in vitro and in vivo in animals [16,17,18,19,20] on the effects of pectin on fecal metabolite profiles. Some of these metabolites, so-called volatile organic compounds (VOCs) [24], are also present in exhaled breath and they have shown distinct profiles in health and disease states [25], e.g., in patients with GI diseases, like irritable bowel syndrome (IBS) [26]. The exhaled VOC profiles have been associated with intestinal microbial composition [27] and they can be affected by major dietary changes [28]. However, data on the impact of pectin supplementation on VOC profiles are currently lacking. The varying effects on microbiota composition and/or fecal SCFA levels are likely due to different methodologies [29], and differences in dosage [19], chemical structure [30], and/or source (e.g., from lemon, apple or sugar beet) [17,20] of pectin used.

Sugar beet pectin, which can be produced from sugar beet pulp as a byproduct in sugar beet industry, received much attention as a potential health promoting food and feed ingredient in the recent years. Sugar beet pectin as compared to citrus and apple pectin, for example, comprises the acetylation of homogalacturonan. A rat model assessed the health effects of sugar beet pectin supplementation in comparison with low- and high-methyl esterified citrus pectin and soy pectin, respectively [17]. Low-methyl esterified citrus pectin and soy pectin significantly increased the concentrations of total SCFA, and of propionate and butyrate in the cecum, whereas sugar beet pectin supplementation led to a stronger increase in the relative abundance of *Lactobacillus* and Lachnospiraceae [17]. Furthermore, in the TIM-2 in vitro colon fermentation model, the propionate production was higher when sugar beet pectin was added in comparison to citrus fruits derived pectin [31]. In addition, it has been reported that sugar beet pectin derived galacturonide oligosaccharides demonstrated prebiotic potential through promoting anti-inflammatory commensal bacteria in the human colon based on an in vitro model using bacterial and host cell cultures [32]. Therefore, the next step would be to investigate whether sugar beet pectin consumption also beneficially impacts the microbiota in vivo in humans. Beneficial modulation of the intestinal microbiota is especially important in people who are prone to developing intestinal problems, such as the elderly. The intestinal microbiota of this group was previously shown to have a lower saccharolytic capacity [7]. A decrease in saccharolytic fermentation and consequently an increased proteolytic fermentation, is considered to be less desired for optimal gut homeostasis, as this is associated with the production of potentially toxic metabolites, such as phenolic and sulfide-containing compounds [33]. Therefore, with this study, we compared fecal microbiota composition, fecal SCFA profiles, and VOCs in the exhaled breath of young adults versus elderly, and to investigate the impact of four weeks sugar beet pectin supplementation on these parameters. We hypothesized that the intestinal microbiota and metabolite profiles in feces and breath differ between young adults and elderly, with a greater response to four weeks pectin supplementation in elderly versus young adults.

## 2. Materials and Methods

### 2.1. Study Overview

This study was part of a larger project on the effect of pectin on GI function [34]. This study was designed as a randomized, double-blind, placebo-controlled, parallel study (Figure S1), which has been approved by the Medical Ethics Committee of the University Hospital Maastricht and Maastricht University (azM/UM, The Netherlands), and it has been registered in the US National Library of Medicine (http://www.clinicaltrials.gov, NCT02376270). It was performed according to the Declaration of Helsinki (latest amendment in Fortalesa, Brasil, 2013) and Dutch Regulations on Medical Research involving Human Subjects (1998) at the Maastricht University Medical Center+ (MUMC+) between March 2015 and April 2016. All of the participants gave written informed consent prior to participation.

### 2.2. Participants

Healthy young adults (18–40 years) and healthy elderly (65–75 years) with a body mass index between 20 and 30 kg/m^2^ were recruited by public advertisements. The key exclusion criteria included GI diseases, abdominal surgery interfering with GI function, use of nonsteroidal anti-inflammatory drugs, and/or vitamin supplementation within 14 days prior to testing, administration of pro-, pre-, or antibiotics in the 90 days prior to the study, pregnancy, lactation, smoking, and history of side effects towards intake of prebiotic supplements. Medical doctor checked other medications use. The sample size calculation was based on a previous study in which the effects of five weeks dietary fiber-enriched pasta intake was investigated [35]. For the sample size calculation, data of the primary study outcome parameter of the original research protocol, intestinal permeability (not included in this manuscript), were used. The sample size calculation showed that each age group should contain at least 48 completers (i.e., 24 per intervention group).

### 2.3. Dietary Intervention

Each subject was randomly assigned to the pectin or placebo group (Figure S1). A person that was not involved in the study generated the list of random allocations while using a computerized procedure. Subjects in the intervention (pectin) group received 15 g/day of pectin (GENU^®^ BETA pectin, CP Kelco, Grossenbrode, Germany). GENU^®^ BETA pectin is a high ester pectin that is extracted from sugar beet pulp, with a degree of acetation of the homogalacturonan backbone of the pectin of the pectin of 18–26%, and molecular weight > 60,000 Da. Subjects in the placebo group received 15 g/day maltodextrin (GLUCIDEX^®^ IT 12, Roquette Freres, Lestrem, France). Maltodextrin and pectin were both supplemented as dry powders free from off-flavors and odors, and packed in closed sachets of a single dose of 7.5 g. The subjects were asked to ingest the supplements twice daily for four weeks, before breakfast and before diner, respectively. Prior to consumption, the content of a sachet was transferred into a glass and mixed with flavored syrup (Karvan Cévitam^®^, Koninklijke De Ruijter B.V., Zeist, the Netherlands) and approximately 200 mL of tap water. Time of consumption was recorded in a diary, and empty and remaining sachets were returned to the investigator to check for product intake compliance. During the intervention periods, all of the subjects were asked to maintain their habitual diet.

### 2.4. Fecal Samples and Microbiota Profiling

Fecal samples were collected before and after the intervention period and immediately stored at −20 °C in home freezers before being transported frozen to the study site. Microbiota composition was determined by sequencing of barcoded 16S ribosomal RNA (rRNA) gene amplicons while using Illumina Hiseq2500 (2 × 150 bp).

DNA was isolated using Repeated-Bead-Beating [36] and purified using the Maxwell^®^ 16Tissue LEV Total RNA purification Kit Cartridge (XAS1220). The V5–V6 region of 16S rRNA gene was amplified in triplicate using primers BSF784/R1064 and fecal DNA as template [37]. Each 35 µL reaction contained 0.7 µL 20 ng/μL DNA template, 7 µL 5 × HF buffer (Thermo Fisher Scientific, Vilnius, Lithuania), 0.7 µL of 10 mM dNTPs (Thermo Fisher Scientific), 0.35 µL DNA polymerase (2 U/µL) (Thermo Fisher Scientific), 25.5 µL nuclease free water (Promega, Madison, WI, USA), and 0.7 µL 10 µM of sample-specific barcode-tagged primers [37]. Cycling conditions were as follows: 98 °C for 30 s, followed by 25 cycles of 98 °C for 10 s, 42 °C for 10 s, 72 °C for 10 s, with a final extension of 7 min. at 72 °C. Subsequently, the triplicate PCR products were pooled for each sample, purified with the CleanPCR kit (CleanNA, The Netherlands), and quantified while using the QubitTM dsDNA BR Assay kit (Invitrogen by Thermo Fisher Scientific, Eugene, OR, USA). In total, we obtained 16S rRNA gene amplicons from 196 fecal samples, eight biological replicates, plus six synthetic microbial communities, which served as a positive control to control for replicability and reflection of the actual composition by the sequencing approach, respectively [37]. An equimolar mix of purified PCR products was prepared and sent for sequencing (GATC-Biotech, Konstanz, Germany, now part of Eurofins Genomics Germany GmbH). Raw sequence reads were subsequently processed while using NG-Tax [37]. The sequencing data are available at the European Nucleotide Archive with accession number PRJEB31775.

### 2.5. Fecal Metabolite Profiling

SCFAs were measured in the feces due to their correlation with a healthy (distal) colon. In addition, we also measured BCFAs (branched chain fatty acids), since their formation indicates protein fermentation instead of only glycosidic fermentation. Concentrations of SCFAs and BCFAs were determined in duplicate. Between 200–300 mg feces were dissolved in 1.0 mL distilled water, mixed, and centrifuged (30,000× *g* for 5 min). Standard solutions of acetic acid, propionic acid, butyric acid, valeric acid, isovaleric acid and isobutyric acid were prepared in concentrations of 0.01–0.45 mg/mL. Two hundred fifty microliters of internal standard solution (0.45 mg/mL 2-ethylbutyric acid in 0.3 M HCl and 0.9 M oxalic acid) was added to 500 µL of the standard solutions and centrifuged samples. After mixing and centrifugation, 150 µL supernatant was used for analysis. SCFAs were quantified while using gas chromatography (Focus GC, Thermo Scientific, Waltham, MA, USA) coupled with a flame ionization detector (FID) (Interscience, Breda, The Netherlands). One μL was injected into a CP-FFAP CB column (25 m × 0.53 mm × 1.00 μm, Agilent, Santa Clara, CA, USA). The initial oven temperature was 100 °C, increased to 180 °C at 8 °C/min., held at this temperature for 1 min, increased to 200 °C at 20 °C/min, and held at this temperature for 5 min, respectively. Injection was done at 200 °C with flow rate of 40 mL/min. at a constant pressure of 20 kPa. Data were processed using Xcalibur^®^ (Thermo Scientific, Waltham, MA, USA). SCFA concentrations were expressed per gram dry matter to correct for the potential impact of stool consistency (potentially altering with aging and by prebiotic intake). Dry matter content was determined by vacuum drying of 500 mg feces for five hours at 60 °C while using a concentrator plus (Eppendorf, Hamburg, Germany).

### 2.6. Volatile Organic Compounds Profiling

Exhaled air samples were collected by breathing into a 3 L Tedlar bag (SKC Limited, Dorset, UK) and being transferred within one hour to carbon-filled stainless steel absorption tubes (Markes International, Llantrisant, UK) while using a vacuum pomp (VWR international, Radnor, PA, USA). VOCs were measured using thermal desorption gas chromatography time-of-flight mass spectrometry (GC-*tof*-MS, (Markes International, Llantrisant, UK), as described previously [38]. Briefly, samples containing VOCs were injected in the system with split ratio 1:2.7. Approximately 40% of the sample was trapped into the cold trap at 5 °C to concentrate the sample. The remaining amount of the sample was stored to the sorption tube. The VOCs in the cold trap were released into a capillary GC column (RTX-5ms, 30 m × 0.25 mm 5% diphenyl, 95% dimethylsiloxane, film thickness 1 m, Thermo Electron TraceGC Ultra, Thermo Electron Corporation, Waltham, MA, USA). The temperature of the GC was programmed, as follows: 40 °C for the first 5 min., and then increased to 270 °C at 10 °C/min. Compounds in the samples were detected by *tof*-MS Thermo Electron Tempus Plus time-of-flight mass spectrometer, Thermo. Electron Corporation, Waltham, MA, USA). Electron ionization mode was set at 70 eV and the mass range 35–350 m/z was measured. The resulting breath-o-grams were denoised, baseline corrected, aligned, normalized by probabilistic quotient normalization, and then scaled for further analyses [39].

### 2.7. Statistical Analyses

Statistical analysis of baseline characteristics of study participants was performed using IBM SPSS Statistics for Windows (version 25.0, Armonk, NY, USA: IBM Corp.). Differences in age and BMI between all young adults and elderly, or between placebo group and pectin group, were shown as means ± standard deviation (SD) and tested using T-tests. The differences in categorical variables were shown as percentages and tested with Chi-square tests or Fisher’s exact tests when appropriate. Baseline samples were used to compare microbiota composition and metabolite profiles (i.e., fecal SCFAs and exhaled VOCs) of young adults to those of elderly individuals, while the impact of pectin supplementation was studied by comparative analysis of pre- and post-intervention samples that was based on intention-to-treat analysis. *p*-values ≤ 0.05 (two-sided) were considered to indicate statistical significance.

Complex data, including microbiota and VOCs, were analyzed using multivariate statistics. Sequence read counts were normalized to microbial relative abundance, and microbiota diversity indices (Faith’s phylogenetic diversity (PD) and inverse Simpson) were calculated at amplicon sequence variant (ASV) level, as implemented in the *Picante* [40] and *Phyloseq* [41] packages, respectively. Wilcoxon test was applied to determine whether diversity as well as relative abundance of specific bacterial taxa, were significantly different between groups since the data was non-parametric. False discovery rate (FDR) was used to correct for multiple testing according to the Benjamini–Hochberg procedure. Unpaired tests were used to determine the differences between age groups at baseline. Paired tests were used to compare pre- vs. post-intervention effects. Pairwise weighted Unifrac (WU) [42] and unweighted UniFrac (UU) [43] distance based principle coordinate analysis (PCoA) was used to visualize microbial community variation at the ASV level [44]. Permutational multivariate analysis of variance (PERMANOVA) was used to test for significant differences between groups, as implemented in the *Vegan* [45] package. Random Forest (RF) analysis (500 trees with four-fold cross validation) was performed to validate the findings of PCoA coupled with PERMANOVA (data not shown), i.e., testing whether microbiota profiles could predict the age group differences and intervention effect. All microbiota based statistical analysis was performed in R (R-3.5.0) [46]. The R code for the analysis is available at GitHub (https://github.com/mibwurrepo/Pectin-elderly-intervention).

Exhaled breath data was analyzed with Principal Component Analysis (PCA) and RF. Data were log transformed to account for data skewed distribution and pareto-scaled to ensure equal contribution of each volatile metabolite in breath in the consequent analysis. RF analysis (with 1000 trees) was performed to discover whether VOCs in exhaled breath could predict the intervention in elderly and young adults, as well as to investigate whether exhaled breath metabolites were different between young and elderly adults. In order to represent the unbiased prediction error, the data was randomly divided into a training- and a validation set. The training set was used to find discriminatory VOCs and to build the classification model. The performance of the RF classification model was demonstrated by the area under the curve of receiver operating characteristic (AUROC) for the validation set. The final results were visualized in a PCA score plot while using the most discriminatory VOCs that were selected in at least 80% of RF iterations in the training set. Statistical analyses of VOCs were performed while using Matlab 2018a (The MathWorks, Natick, 2018).

SCFAs were single parameters and analyzed with univariate statistics. Independent-samples T Tests were performed to compare the SCFA levels of young adults versus elderly. Unstructured linear mixed model analyses were performed to compare SCFA levels within age groups and between intervention groups. Individual was included as random factor. Intervention group, time and ‘intervention group × time’ were included as fixed factors, and corrections for baseline values were made. Statistical analyses of SCFAs were performed while using IBM SPSS Statistics for Windows (version 25.0, Armonk, NY, USA: IBM Corp.)

## 3. Results

### 3.1. Subjects

52 healthy young adults and 48 elderly were included for the current study, of whom the baseline characteristics are provided in Table 1. Elderly had a significantly higher age, body mass index (BMI), and medication use when compared with young adults. Placebo and pectin groups did not differ for any of the baseline characteristics in either of the two age groups. Three young adults (i.e., two in the pectin group, one in the placebo group) dropped out during the study due to overt non-compliance or the prescription of antibiotic therapy. From these drop-outs, samples were used for baseline characteristics and fecal- and exhaled breath analyses, but were not included in the post intervention measurements. DNA isolation failed for one fecal sample from a young adult (placebo group, post intervention), and hence was excluded for microbiota profiling.

### 3.2. Young Adults and Elderly Showed Similar Fecal Microbiota Composition, SCFA- and Exhaled VOC Profiles

**Figure 1:**
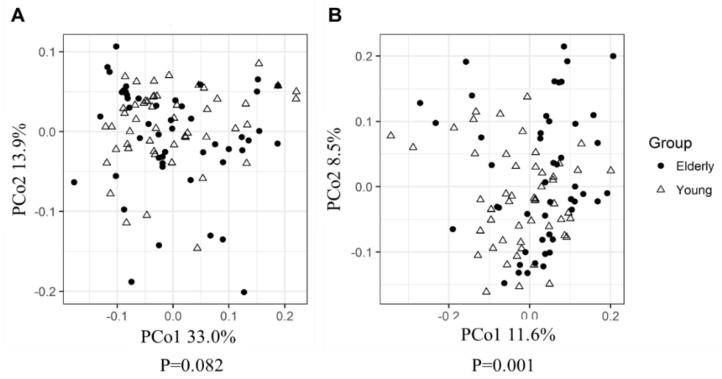
Baseline principle coordinate analysis (PCoA) plots based on weighted UniFrac (**A**) and unweighted UniFrac (**B**) pairwise distance matrices using amplicon sequence variant-level data, show overlapping microbiota profiles of young adults and elderly. Significance of observed differences between groups was evaluated by PERMANOVA.

**Figure 2:**
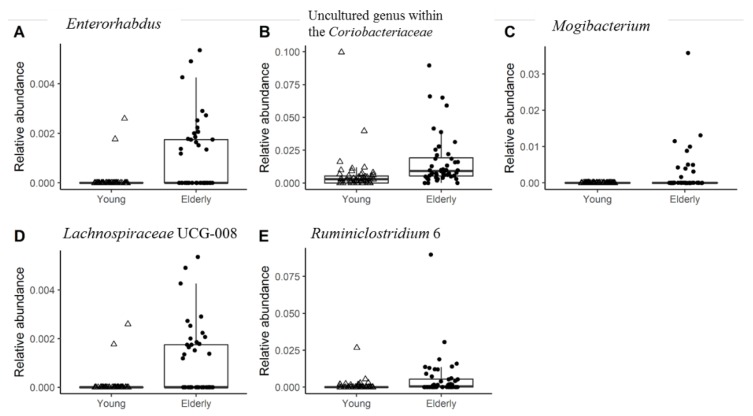
Genus level taxa that significantly differed (false discovery rate (FDR) < 0.05) in relative abundance between young adults and elderly at baseline. The relative abundance of each genera are shown as follows, (**A**) *Enterorhabdus*; (**B**) Uncultured genus within the *Coriobacteriaceae*; (**C**) *Mogibacterium*; (**D**) *Lachnospiraceae* UCG-008; (**E**) *Ruminiclostridium* 6.

PCoA that was based on weighted UniFrac (taking relative abundance of bacterial ASVs into account) revealed no significant differences between the microbiota of young adults and elderly (Figure 1A). However, PCoA based on unweighted UniFrac distances (only taking into account presence/absence of bacterial ASVs, placing emphasis on less abundant species), did show a small though significant difference between the microbiota of young adults and that of elderly (*p* = 0.001), with 2.4% of microbiota variation being explained by age groups (Figure 1B). The RF analysis to determine the differences in microbiota profiles between young adults and elderly showed an out-of-bag error rate of 29.29%, which indicated relatively small differences in microbiota profiles. The relative abundances of five genus-level taxa (*Enterorhabdus, Ruminiclostridium* 6*,* uncultured genus within the *Coriobacteriaceae, Mogibacterium, Lachnospiraceae* UCG-008) out of 224 genera were significantly different between young adults and elderly before the intervention (Figure 2). In addition, no significant differences were found in their fecal microbiota alpha diversity at baseline (Figure S2). Furthermore, in both age groups, PERMANOVA analysis of microbiota profiles based on weighted UniFrac and unweighted UniFrac distance matrices showed no significant difference between the placebo and pectin supplementation groups at baseline.

Baseline fecal SCFA and BCFA concentrations revealed no significant differences between young adults and elderly (Table 2). Independent of age group, large individual differences were found for all SCFAs, as indicated by the relatively high SD.

**Figure 3:**
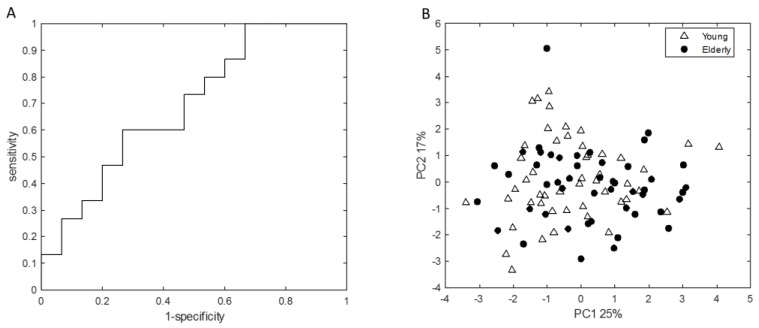
(**A**) Receiver operating characteristic curve performed on the validation set, with area under the curve = 0.70. (**B**) Principal Component Analysis (PCA) score plot, performed on a set of 15 VOCs that were found important (set of the most discriminatory volatile organic compounds (VOCs) selected in at least 80% of RF iterations) for classification in the Random Forest (RF) model, showing no clear groupings in exhaled breath profiles between young adults and elderly. Percentages given at both axes indicate percentage of variation explained by either principal component.

The VOC-based RF analysis using a set of 15 VOCs to determine the differences in exhaled VOCs between young adults and elderly at baseline showed an AUROC of 0.70 with sensitivity and specificity of 0.6 and 0.58 in the validation set (Figure 3A), which indicated relatively small differences in exhaled breath profiles, which is in line with fecal microbiota and SCFA data. PCA analysis performed on the VOCs that were important for classification in the resulting RF model showed no clear differences between young adults and elderly (Figure 3B). This is in accordance with the PCA analysis performed on the complete breath profiles (Figure S3).

### 3.3. Four Weeks of Sugar Beet Pectin Supplementation Did Neither Alter Fecal Microbiota Composition, Nor SCFA- and Exhaled VOC Profiles

**Figure 4:**
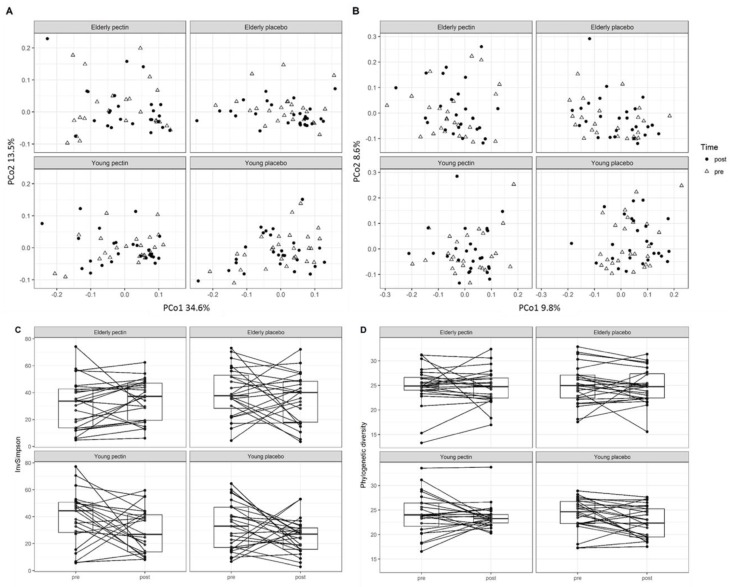
Intervention effects on microbiota composition and alpha diversity in young adults and elderly. PCoA plots at baseline and after four weeks sugar beet pectin or placebo supplementation based on weighted UniFrac (**A**) and unweighted UniFrac (**B**), showed no clear groupings in microbiota profiles between pre- and post-intervention. (**C**) Comparison of phylogenetic diversity and (**D**) InvSimpson indices pre- vs. post-intervention at individual level, showing no significant changes in microbial diversity pre- vs. post-intervention. Significance of differences between groups was evaluated by PERMANOVA.

Comparative analysis between the pre- and post-intervention samples did not reveal any significant effects of pectin supplementation on global microbiota profiles at ASV level (Figure 4A, B) and in-depth microbial composition (i.e., detailed taxa comparison), or impact on microbial phylogenetic diversity (Figure 4C) and InvSimpson diversity indices (Figure 4D). In addition, we did observe significantly smaller intra-individual variation over the treatment period when comparing to inter-individual variation, based on weighted and unweighted UniFrac (Table S2). Interestingly, the young pectin group showed significantly decreased inter-individual variation in phylogenetic diversity post pectin treatment, while the other groups displayed a more heterogeneous response. Four (except for *Ruminiclostridium* 6) out of five genera that were different before the intervention remained significantly different between age groups after the intervention (Figure S4), which suggested that these differences are consistent between elderly and young adults. In terms of subjects who were shown to have a higher relative abundance of corresponding taxa after the intervention, 72.0% (*Enterorhabdus*), 91.5% (*Coriobacteriaceae* uncultured), 46.2% (*Lachnospiraceae* UCG-008), and 90.9% (*Mogibacterium*) were the same subjects as before the intervention. These differences in bacterial relative abundance could not be explained by medication use or other characteristics that were noted at baseline (Table S1).

Four weeks of sugar beet pectin intake also did not significantly change fecal SCFA or BCFA concentrations in young adults, or in elderly (Table 3). In addition, within exhaled breath, several SCFAs were detected, namely acetic acid, pentanoic acid, propionic acid, and 2-methyl-propanoic acid, which did not change after the intervention.

**Figure 5:**
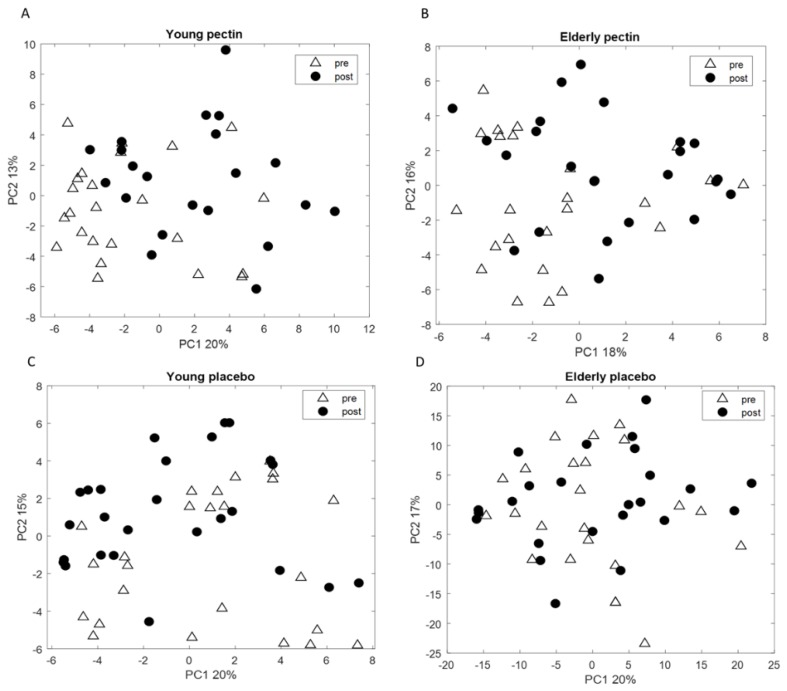
PCA score plot based on the set of (**A**) 11 volatile metabolites in the exhaled breath of young adults to discriminate between pre and post-pectin intervention; (**B**) 12 volatile metabolites in the exhaled breath of elderly to discriminate between pre and post-pectin intervention. PCA score plots performed on the set of 14 VOCs measured in exhaled breath of (**C**) young adults; and on 16 VOCs measured in exhaled breath of (**D**) elderly; for pre- and post-placebo intervention. No groupings of the samples are observed. Discriminatory VOCs were selected in at least 80% of RF iterations.

RF analysis was performed between pre- and post- pectin intervention data for young adults and elderly separately in order to investigate the effect of pectin on the VOC profiles of exhaled breath. The performance of the model, based on the most discriminatory VOCs in breath, resulted in an AUROC of 0.57 and 0.50 for the validation set for young adults and elderly, respectively, which indicated that samples that were taken before and after the-intervention did not differ. The corresponding PCA score plots were performed on sets of 11 and 12 VOCs for young adults and elderly, respectively, as these were the most discriminatory compounds that were selected in at least 80% of RF iterations (Figure 5A,B). No clear groupings were found between post and pre-intervention, indicating similarity in the breath profiles.

The RF models for the placebo intervention showed AUROCs of 0.32 and 0.40 for young adults and elderly, respectively. The PCA score plots shown in Figure 5C,D indicate that similarly to pectin, placebo did not alter the VOC profiles in exhaled breath in either of the age groups.

In addition, VOCs of young adults and elderly were compared between pectin and placebo supplementation at baseline and post-intervention. The implemented RF models, separated between young adults and elderly, revealed no predictive power, which indicated similar breath profiles of placebo and pectin supplementation at baseline (AUROC of 0.35 for both models). The post-intervention RF classification model led to AUROCs of 0.34 and 0.58 for the validation set for young adults and elderly, respectively, demonstrating no differences in the breath profiles between placebo and pectin post-intervention.

Together with the observations on microbiota composition, SCFAs and VOCs, this suggests that in this study, pectin had no significant impact on the fecal microbiome, or on breath metabolite profiles, either in elderly nor in young adults.

## 4. Discussion

In the present study, we compared healthy young adults versus healthy elderly and studied the effect of sugar beet pectin supplementation on fecal microbiota composition, fecal SCFA, and exhaled breath VOC profiles. We hypothesized that intestinal microbiota and metabolite profiles in feces and breath differ between elderly and young adults. We did observe limited and very subtle differences between age groups with respect to microbiota composition, with only five out of 224 genera being significantly higher in relative abundance in elderly as compared with young adults. No significant differences were found in fecal SCFA and exhaled VOC profiles between the age groups. In addition, in neither of the two age groups were any effects of pectin supplementation on fecal microbiota, SCFA, and exhaled VOC profiles observed.

Aside from the small differences in the composition of the intestinal microbiota between the age groups, microbiota composition, and its activity in the healthy elderly was comparable with profiles in the healthy young adults. This suggests that health status, rather than chronological age, might affect microbiota composition and activity, an observation that is in line with findings in previous studies [3]. Biagi et al. [47] compared the microbiota of young adults with that of non-institutionalized elderly with good physical and cognitive health status and also demonstrated a high similarity between young and elderly. Jackson et al. [48], specified the health status (i.e., frailty level) of recruited community dwellers according to the Rockwood frailty index, and revealed an association between microbiota profile (e.g., decrease in microbial diversity) and increased frailty. Claesson et al. [49] classified elderly into four different groups (i.e., community dwellers, outpatients, short-term hospitalized, and long-term hospitalized) and demonstrated that changes in residency (e.g., changing from community dwellers to long-stay), which suggests differences in health status, which are correlated with dietary intake patterns. This difference in food intake could contribute to perturbations in the microbiota composition and/or microbial activity [49]. Specifically, the long stay subjects showed decreased acetate, propionate, valerate, and butyrate levels as compared to community dwellers [49]. This was further confirmed by functional analysis, which showed that institutionalized elderly [50] and elderly using medication [7] had a decreased number of genes coding for SCFAs production in their microbiota when compared with young adults.

Five genera (*Enterorhabdus, Ruminiclostridium* 6*, Coriobacteriaceae* uncultured*, Mogibacterium, Lachnospiraceae* UCG-008) were significantly higher in relative abundance in the fecal microbiota of elderly, as compared to that of young adults. *Mogibacterium* spp. have previously been isolated from oral cavities [51] and the prevalence of dental caries is higher in the elderly [52,53]. Moreover, one recent study employing metagenomic sequencing showed the translocation of oral microbes to the intestine [53]. Nevertheless, the role of *Mogibacterium* in the intestine remains unclear. The aerotolerant genus *Enterorhabdus* was previously shown to have a higher relative abundance in prediabetic subjects, when compared to healthy controls [54]. Moreover, the increased prevalence of prediabetes was associated with higher BMI [55]. This is confirmed in the present study, as the BMI of elderly was significantly higher than that of young adults while the relative abundance of *Enterorhabdus* was also increased in the elderly. *Ruminiclostridium* 6, *Coriobacteriaceae* uncultured and *Lachnospiraceae* UCG-008 are not well classified genus-level groups, up to now. In addition, subjects maintained their habitual diet during the study. It cannot be ruled out that possible confounders, such as differences in habitual diet or other lifestyle factors, have contributed to the minor differences between the microbiota of young adults and elderly in the current study.

Pectin supplementation did not affect fecal microbiota, SCFA and exhaled VOC profiles in elderly, nor in young adults, respectively. Interventions designed to study the effects of non-digestible carbohydrates on microbiota composition and/or activity in elderly, so far mainly focused on inulin [56], FOS [9], GOS [12], trans-galactooligosaccharide mixture (B-GOS) [10,11], and a non-digestible carbohydrate mixture (of resistant starch, GOS, corn fiber, polydextrose and wheat dextrin) [57]. In all studies bifidogenic effects were demonstrated, but only two studies reported changes in microbial activity, i.e., increase in lactic acid [11] and butyrate [12] levels, when B-GOS or GOS was provided, respectively. Studies investigating the effects of pectin on the intestinal microbiota have been based on both in vitro systems [14,15,23], in vivo models [16,17,18,19,20], and in humans [21,22], demonstrating increases in SCFA levels and/or alteration in microbial composition. One human intervention study with 24 g/day pectin (unspecified origin) in constipated adults showed significant increases in fecal *Bifidobacterium* and *Lactobacillus* levels, as well as a significant decrease in *Clostridium* [22]. However, in the present study, pectin supplementation did not affect fecal microbiota composition. Differences with the present study could in part be explained by differences in the source, chemical structure, and/or amount of pectin supplemented (15 g/day present study vs. 24 g/day), as well as differences in health status (e.g., constipated adults have relatively long residence time in colon).

In line with the present study, no bifidogenic effect was observed, when the same sugar beet pectin was supplemented to rats for seven consecutive weeks continuously [17]. The duration of the present study was even shorter (i.e., four weeks) when compared to the above rat study, which might have also impacted on potential intervention effects. It has previously been shown in in vitro fermentation studies with human fecal microbiota that an increased degree of esterification decreased pectin fermentation rate [58], and the production of SCFAs was found to be decreased in the cecum of conventional rats (rats colonized with rat fecal material) [30]. Consistent with our current study, the rat model demonstrated that the sugar beet pectin did not affect SCFA profiles in cecum or in colon, except for a significantly decreased propionate level in the colon [17]. To this end, it should also be noted that metabolites produced in the gut lumen are known to be readily absorbed and transported to different compartments of our body, after which a proportion of the metabolites will be exhaled by the lungs and thereby detected in breath.

Recent studies have shown that VOC profiles in exhaled air have diagnostic potential [26,59,60,61]. It has been previously demonstrated that exhaled VOCs showed a very strong correlation with intestinal microbiota composition as studied in patients with Crohn’s disease [27], but also in IBS [26]. Therefore, exhaled VOCs can also be used as an indicator of intestinal microbiota activity, either by their direct metabolic activity or by conversion of metabolites derived from host processes. In the studies of Blanchet et al. [62] and Dragonieri et al. [63], the effect of age on exhaled metabolic breath profiles was investigated while using two different analytical methodologies, i.e., mass spectrometry and the electronic nose, respectively. In both studies the effect of age on VOCs profiles was very limited. In the study by Blanchet et al., the VOCs profiles have been found to be statistically significant between age ranges divided in segments of ten years. Although the VOCs profile was statistically significant between those age segments, the overall effect was not strong enough to lead to a discriminatory model. In the similar study by Dragonieri et al., an exhaled breath profile of young (below 50 years old) and older individuals showed no differences while using canonical discriminant analysis. This is in accordance with the present study, where healthy young adults and elderly showed high similarity in exhaled VOC profiles in line with the microbiota profiles. Several investigators have pointed to the effects of dietary nutrients on VOC profiles of the exhaled breath both in clinical and animal studies [26,64,65,66]. The changes in exhaled breath composition due to dietary nutrients have been related to their direct impact on metabolism and/or because they modify the intestinal microbiota (composition and/or activity). In a recent study by Smolinska et al., significant differences in exhaled VOC profiles of adults were observed 240 min after consuming two infant formula diets that only differed with respect to lipid structures, showing that differences in dietary nutrients can lead to short term changes in exhaled breath composition [67]. Although pectin is a dietary fiber that could potentially alter VOC profiles by increasing the intestinal metabolite production, in the current study no intervention effect was shown on the exhaled VOC profiles of young adults and elderly. This is in contrast to a study by Raninen et al. [68], which investigated the level of 15 VOCs in exhaled breath of subjects that consumed either a high fiber diet (44 g/day of whole grain rye) or a low fiber diet (17 g/day of whole grain rye) and demonstrated significant differences in the VOC profiles. In addition, a single test meal (mixture of different carbohydrates) also affected exhaled VOC profiles. Observed differences between studies might be explained by different types (cereal vs. fruit or vegetable source) and/or dosages of fibers used.

## 5. Conclusions

In this study, aside from the subtle differences in microbiota composition, healthy young adults and healthy elderly showed similar profiles in microbiota composition and microbial activity, as well as the breath metabolite profiles at baseline. These findings are in line with our recent understanding that the microbiota composition and activity are preserved in healthy aging and changes are primarily due to alterations in health status and lifestyle factors [3]. In addition, no effects of pectin supplementation on microbiota composition, fecal SCFA- or breath metabolite profiles were observed, which indicated resilience towards pectin exposure. It would be interesting to investigate the effects of pectin in more susceptible subgroups of elderly (i.e., frail, or with specific comorbidities). For future research, studies investigating the dynamics of intestinal microbial composition, activity, and exhaled VOC profiles under different health conditions, as well as how they response to different dietary fiber supplementations, are warranted.

## Figures and Tables

**Table 1:** Baseline characteristics of the young adults (*n* = 52) and elderly (*n* = 48) study populations.

	Young Adult (*n* = 52)			Elderly (*n* = 48)			All Young Adults vs. All Elderly
	Placebo (*n* = 27)	Pectin (*n* = 25)	*p*-Value	Placebo (*n* = 24)	Pectin (*n* = 24)	*p*-Value	*p*-Value
Age (years)	22.8 ± 4.1	23.4 ± 4.5	0.614	69.8 ± 2.4	69.5 ± 3.2	0.723	<0.001
Female (%)	48.2	68.0	0.148	50.0	37.5	0.383	0.164
BMI (kg/m^2^)	22.6 ± 2.7	23.2 ± 2.7	0.444	26.2 ± 2.8	25.5 ± 2.6	0.334	<0.001
Medication (%)	0	0	1.000	33.3	45.8	0.376	<0.001
PPI (%)	0	0	1.000	12.5	12.5	1.000	<0.001
Statins (%)	0	0	1.000	4.2	4.2	1.000	<0.001
Antihypertensives (%)	0	0	1.000	8.3	12.5	0.637	<0.001
Other medication (%)	0	0	1.000	12.5	16.7	0.683	<0.001

Differences in age and BMI between all young adults and elderly, or between placebo group and pectin group, were tested using *T*-tests. Differences in sex (i.e., female or male) were tested with chi-square tests. Differences in medication use were tested by Fisher’s exact tests. Values are presented as mean ± SD or percentage (%). BMI, body mass index. PPI, proton-pump inhibitors.

**Table 2:** Fecal short-chain fatty acid concentrations (µmol/g dry content) of young adults (*n* = 52) and elderly (*n* = 48) at baseline.

	Young Adults (*n* = 52)		Elderly (*n* = 48)		*p*-Value *
	Mean	SD	Mean	SD	
Acetic acid	225.9	187.6	201.6	145.2	0.469
Propionic acid	71.1	66.4	58.1	53.2	0.281
Butyric acid	59.2	45.0	56.6	49.8	0.785
Valeric acid	8.4	6.4	9.3	6.7	0.473
Isobutyric acid	6.8	3.7	7.2	6.0	0.715
Isovaleric acid	10.6	5.6	11.1	9.0	0.729

* Differences between age groups were tested by independent-sample T Tests. SD, standard deviation.

**Table 3:** Fecal short-chain fatty acid concentrations (µmol/g dry content) of placebo- and pectin intervention groups at baseline and after four weeks supplementation, in young adults and elderly.

	Intervention	Young Adults					Elderly				
		Pre-Intervention		Post-Intervention		*p*-Value * (Placebo vs. Pectin)	Pre-Intervention		Post-Intervention		*p*-Value * (Placebo vs. Pectin)
		Mean	SD	Mean	SD		Mean	SD	Mean	SD	
Acetic acid	Placebo	210.2	182.7	263.7	233.3	0.202	167.9	95.0	230.5	188.1	0.548
	Pectin	242.8	195.1	237.8	222.4		235.3	178.0	268.4	155.2	
Butyric acid	Placebo	56.1	41.5	77.5	55.8	0.066	44.1	25.8	56.3	46.8	0.280
	Pectin	62.6	49.1	61.1	47.2		69.2	63.9	67.3	37.5	
Isobutyric acid	Placebo	6.2	2.4	7.9	4.9	0.495	6.1	3.7	7.2	4.3	0.290
	Pectin	7.5	4.8	8.2	5.3		8.3	7.5	7.8	4.0	
Isovaleric acid	Placebo	10.1	3.5	12.4	8.0	0.654	9.6	6.0	10.9	6.2	0.364
	Pectin	11.2	7.2	12.5	8.2		12.7	11.2	11.8	6.4	
Propionic acid	Placebo	71.0	69.6	99.4	131.0	0.074	40.7	18.0	52.6	32.5	0.752
	Pectin	71.2	64.3	66.8	49.0		75.6	69.4	81.8	43.8	
Valeric acid	Placebo	7.1	5.1	10.7	13.5	0.113	7.9	4.3	9.4	7.2	0.391
	Pectin	9.8	7.4	9.6	5.5		10.8	8.3	10.3	4.1	

* Corrected for baseline values. Within age groups, differences between interventions were tested by an unstructured linear mixed model and correction for baseline values. SD, standard deviation.

## Supplementary Materials

The following are available online at https://www.mdpi.com/2072-6643/11/9/2193/s1, Figure S1: Schematic overview of the study design., Figure S2: Fecal microbiota of young adults and elderly did not show significant differences in alpha diversity at baseline., Figure S3: PCA score plot performed on the complete breath profiles of young adults and elderly., Figure S4: Significantly different genus level taxa (FDR<0.05), comparing the microbiota of young adults and elderly after intervention., Table S1: Contribution of participants’ baseline characteristics to baseline microbiota variation., Table S2: Inter- and intra-individual distance over the intervention period.
