# Supplementary material for: Sugar Beet Pectin Supplementation Did Not Alter Profiles of Fecal Microbiota and Exhaled Breath in Healthy Young Adults and Healthy Elderly
Source: Nutrients. 2019 Sep 12;11(9):2193. doi: 10.3390/nu11092193 (PMC6770243; doi:10.3390/nu11092193)
Supplement: Supplementary file 1 [file nutrients-11-02193-s001.pdf]

Supplementary Material:

The following are also available online at www.mdpi.com/xxx/s1:

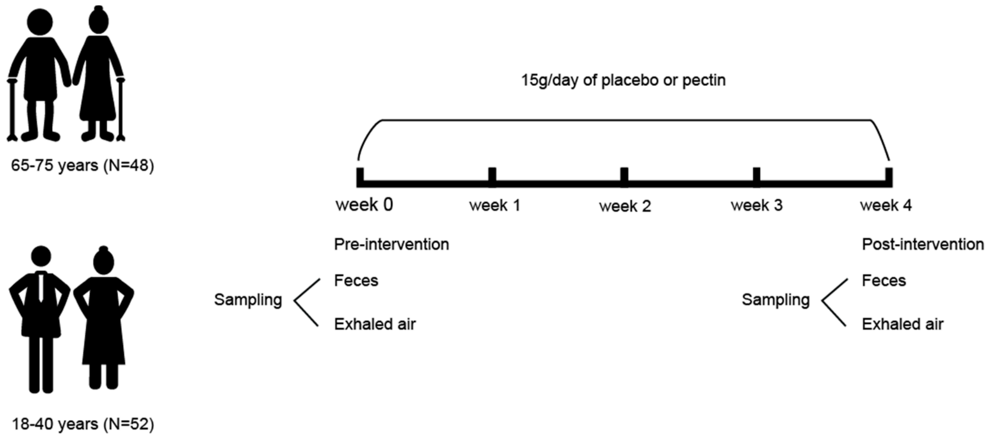

Figure. S1: Schematic overview of the study design. Forty-eight healthy elderly and 52 young adults started this study. Participants consumed either 7.5g pectin or 7.5g maltodextrin (placebo) twice daily for four weeks. Feces and exhaled air were sampled before and after the intervention for analyses.

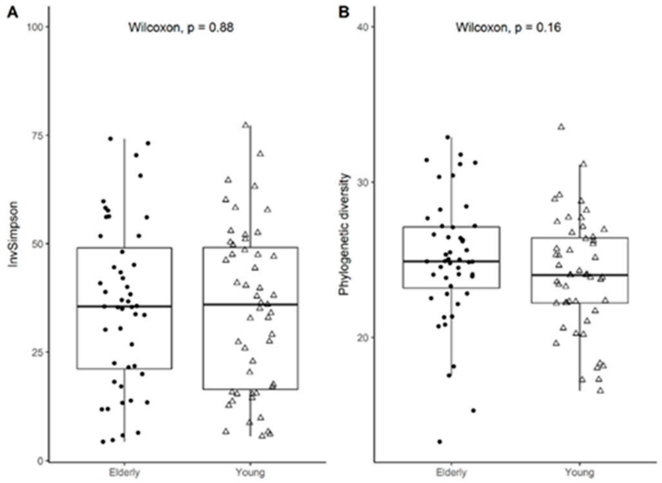

Figure. S2: Fecal microbiota of young adults and elderly did not show significant differences in alpha diversity at baseline. The diversity of the microbiota was evaluated by inverse Simpson's index and phylogenetic diversity.

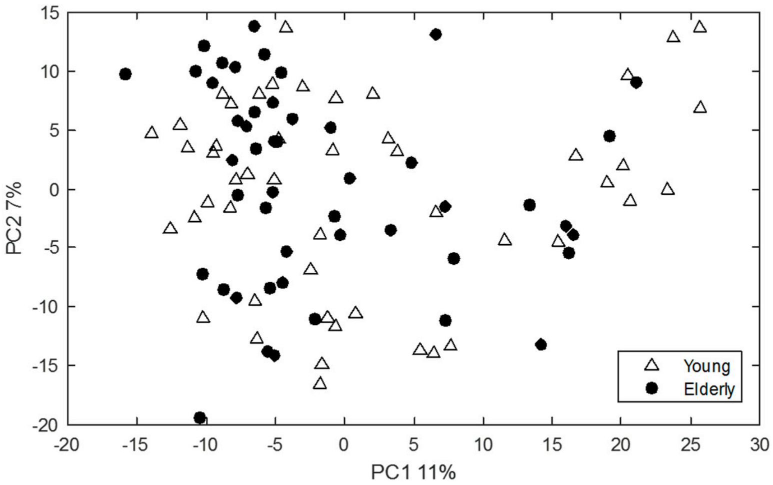

Figure S3: PCA score plot performed on the complete breath profiles of young adults and elderly. No clear groupings in exhaled breath profiles could be observed between young adults and elderly.

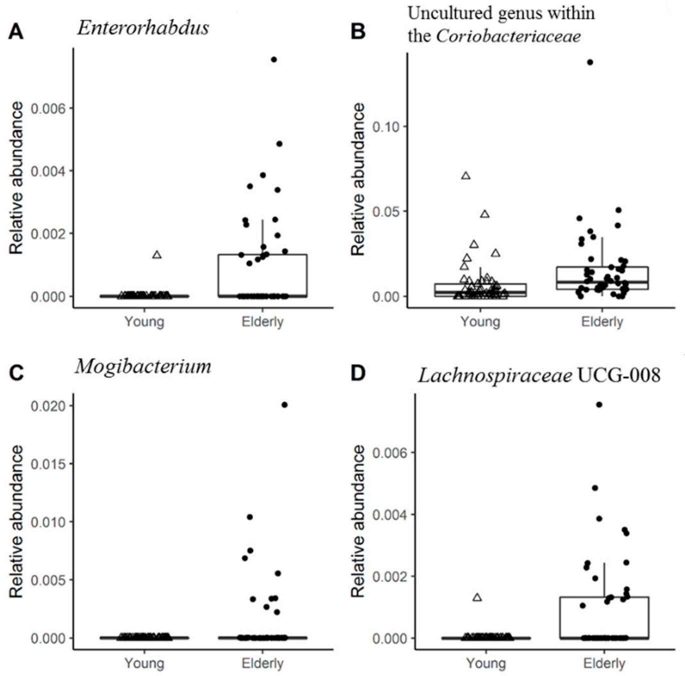

Figure. S4: Significantly different genus level taxa (FDR<0.05), comparing the microbiota of young adults and elderly after intervention.

Table S1. Contribution of participants' baseline characteristics to baseline microbiota variation.

	weighted UniFrac		unweighted UniFrac	
	R-square	P-value	R-square	P-value
Age	0.0141	0.5039	0.0049	0.7895
BMI (kg/m2)	0.0044	0.8098	0.0009	0.9590
Alcohol (units/week)	0.0264	0.2752	0.0271	0.2810
Sport (hours/week)	0.0023	0.8904	0.0003	0.9867
Sex (male/female)	0.0047	0.6284	0.0094	0.4016
Medication (yes/no)	0.0006	0.9349	0.0029	0.7593

Allergy (yes/no)	0.0035	0.6957	0.0024	0.7910
Vegetarian (yes/no)	0.0007	0.9299	0.0240	0.0921
Food supplements (yes/no)	0.0094	0.3805	0.0029	0.7524
Disease history (yes/no)	0.0116	0.3201	0.0197	0.1478

Contribution of baseline characteristics of participants was tested by fitting available variables to the ordination object. The variation of baseline microbiota profiles could not be explained by any of the included baseline variables.

Table S2. Inter- and intra-individual distance over the intervention period.

	Inter-individual distance	Intra-individual distance	P-value
Weighted UniFrac	0.21±0.07	0.14±0.07	<0.001
Unweighted UniFrac	0.43±0.07	0.24±0.09	<0.001

Differences between inter-individual distance and intra-individual distance, were tested using T-tests. Values are presented as mean ± SD. Higher value in distance indicates smaller similarity.
